# Occupational buckwheat allergy in a health food store employee: From inhalative exposure to anaphylaxis 

**DOI:** 10.5414/ALX02561E

**Published:** 2025-03-28

**Authors:** Julia Felicitas Pilz, Valentina Faihs, Claudia Kugler, Ulf Darsow, Tilo Biedermann, Knut Brockow

**Affiliations:** Department of Dermatology and Allergy Biederstein, School of Medicine and Health, Technical University of Munich (TUM), Munich, Germany

**Keywords:** buckwheat, occupational anaphylaxis, hidden allergen, inhalant allergen

## Abstract

Abstract. Background: Buckwheat allergy is common in East Asian countries with high buckwheat consumption. However, with increasing popularity of buckwheat as a gluten-free food, it is also expected to become more widespread in Europe. Case report: A health food store employee experienced anaphylaxis with urticaria, angioedema, and dyspnea after eating a slice of buckwheat bread. Prior to this reaction, the patient had repeatedly noticed rhinoconjunctival itching and sneezing when handling buckwheat. A positive skin prick test with buckwheat flour and elevated specific IgE levels to buckwheat confirmed the suspected diagnosis. Conclusion: Food industry employees may develop sensitization to buckwheat through inhalation of buckwheat flour. This sensitization can lead to rhinoconjunctival symptoms upon airborne exposure and to anaphylaxis after ingestion.

## Introduction 

Buckwheat is a pseudocereal from the Polygonaceae family [1], known for its versatility as a component in a range of foods like noodles, bread, pancakes, porridge, and tea [2, 3]. While buckwheat is a traditional ingredient in East Asian cuisine, it is also becoming increasingly popular in Europe as a gluten-free and nutritious alternative [2, 3]. 

Countries with high buckwheat consumption, such as Japan, Korea, and China, report the highest prevalence of buckwheat allergy, ranging from 0.1 – 0.4% [4, 5, 6, 7]. Buckwheat has been found to cause sensitization not only through ingestion but also through occupational inhalation exposure [7, 8]. Clinical manifestations of IgE-mediated buckwheat allergy include rhinoconjunctivitis and asthma following airborne contact, as well as contact urticaria or anaphylaxis after ingestion [7]. 

## Case report 

A 70-year-old female patient, who worked at a health food store until her retirement, reported an anaphylactic reaction to buckwheat. A few minutes after consuming a slice of buckwheat bread, she developed angioedema of the hands and tongue, generalized urticaria, and dyspnea. Emergency treatment included systemic antihistamines and corticosteroids. The patient mentioned that, in the years prior to the reaction, she had experienced rhinoconjunctival itching and sneezing while grinding buckwheat at work. 

Following the anaphylactic reaction, the patient strictly avoided consuming buckwheat, as she suspected a buckwheat allergy herself. Later, the store stopped processing buckwheat in-house, eliminating allergen exposure. The patient continued working at the store without experiencing any further allergic reactions. 

## Allergy diagnostics 

A skin prick test revealed a positive reaction to buckwheat (15 mm wheal, 30 mm erythema). Total IgE was 91 kU/L, with specific IgE levels of 3.2 kU_A_/L for buckwheat extract and 0.6 kU_A_/L for the buckwheat single allergen Fag e 2 (2S albumin). IgE levels were determined using the Allergy Explorer ALEX^2^ (Macro Array Diagnostics, Vienna, Austria). 

**Figure 1 Figure1:**
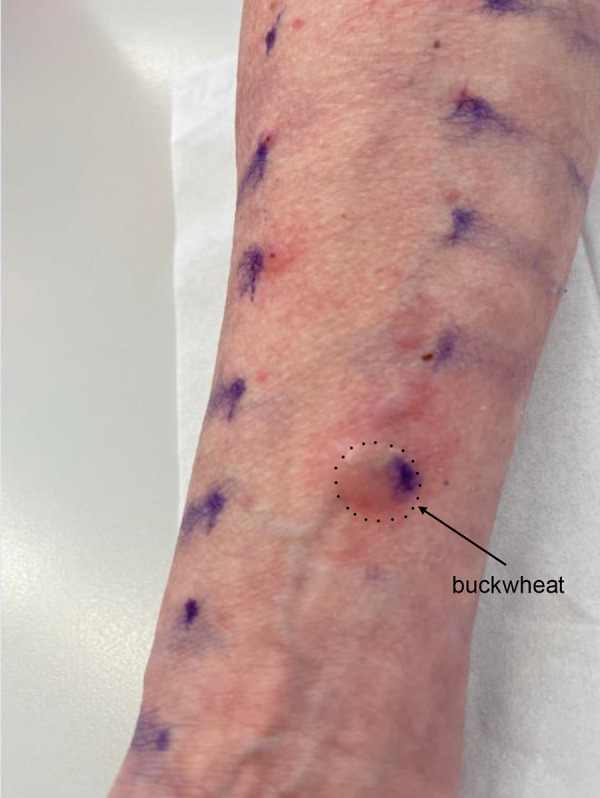
Skin prick test results with buckwheat flour.

Based on the clear sensitization to buckwheat and the patient’s history, an occupational immediate-type allergy to buckwheat was confirmed. [Fig Figure1]

## Discussion 

Exposure to buckwheat can occur during the production or handling of buckwheat-based foods (occupational exposure), ingestion of buckwheat foods (food allergen), or through environmental contact, such as using buckwheat husk pillfows (domestic exposure) [7]. 

The patient’s allergy was caused by occupational inhalation exposure to buckwheat and initially manifested as rhinoconjunctival symptoms. This aligns with other cases reported in East Asia and Europe, where chefs, bakers, and employees in the food and retail industries developed occupational buckwheat allergies, presenting with asthma, rhinitis, conjunctivitis, and/or contact urticaria [7, 8]. In such cases, epicutaneous contact may also be a contributing pathway for sensitization. 

Patients with occupational inhalation exposure to buckwheat are at increased risk of anaphylaxis following consumption of buckwheat products [8]. Anaphylaxis triggered by buckwheat-containing bread, pancakes, noodles, crepes, pizza, porridge, cereal bars, or cakes has been documented, including two fatal cases [3, 7]. It is important to note that after the development of a work-related allergy, buckwheat consumption often occurs outside the occupational context. 

Pillows made from buckwheat husks, initially popular in China and Korea, are now used globally [7]. Following domestic airborne sensitization, these pillows can act as a hidden allergen source, causing nocturnal asthma and rhinitis attacks [7]. 

When advising patients with a buckwheat allergy, it is important to stress that buckwheat may be a hidden allergen, for which there is no labelling requirement in Europe [3]. As a pseudocereal, buckwheat does not cross-react with “true” cereals like wheat. However, clinically relevant cross-reactions with quinoa, coconut, poppy seed, peanut, hazelnut, and latex have been reported in affected individuals [2]. 

## Conclusion 

Buckwheat can cause occupational allergies, particularly in the food and retail industries. While it is becoming increasingly popular in Europe as a gluten-free pseudocereal, it is important to recognize that it may act as a hidden allergen. 

## Authors’ contributions 

Conceptualization: JFP, CK, KB; Data curation: JFP, VF, CK, UD; Formal analysis: JFP, KB; Funding acquisition/resources: TB, KB; Investigation: JFP, VF, CK; Methodology: JFP, KB; Project administration: JFP, KB; Supervision: TB, KB, UD. Visualization: JFP; Writing – Original Draft Preparation: JFP, KB; Writing – Review & Editing: JFP, VF, CK, UD, TB, KB. 

## Funding 

This work was supported by the German Federal Ministry of Education and Research (Bundesministerium für Bildung und Forschung, BMBF) as part of the ABROGATE project (grant number 01EA2106A) awarded to KB. Additional support was provided by the German Research Foundation (Deutsche Forschungsgemeinschaft, DFG) within the RTG 2668 framework to KB and TB. 

## Conflict of interest 

JFP, VF, TB, and KB have received material support from Macro Array Diagnostics AG unrelated to the submitted work. TB has received honoraria for presentations from Alk-Abello, Mylan, and Phadia Thermo Fisher, financial support for clinical trials funded by Alk-Abello, and research funding from Phadia Thermo Fisher and Almirall. KB reports honoraria for presentations and material support for a clinical trial from Thermo Fisher. The authors declare no further relevant conflicts of interest related to the content of this study.
